# Air sampling for detection of infectious laryngotracheitis (ILT) in commercial poultry flocks

**DOI:** 10.1186/s13104-020-05399-2

**Published:** 2020-12-09

**Authors:** Lauren Brown, Dilhani Premaratna, Yonatan Segal, Travis Beddoe

**Affiliations:** 1 Department of Animal, Plant and Soil Science and Centre for AgriBioscience (AgriBio), La Trobe University, Bundoora, Melbourne, VIC Australia; 2 Center for Livestock Interactions With Pathogens (CLiP), La Trobe University, Bundoora, Melbourne, VIC Australia; 3 Department of Jobs, Precincts and Regions, Victorian Government, Attwood, Melbourne, VIC Australia

**Keywords:** Air sampling, ILT, PCR, Gallid herpesvirus 1, Poultry

## Abstract

**Objective:**

Infectious laryngotracheitis (ILT) is an acute and highly contagious viral respiratory disease of poultry, caused by gallid herpesvirus 1 (ILTV), which causes significant economic losses. Due to recent outbreaks of ILT in Australia, it has been proposed that ILT could be transmitted between poultry sheds by airborne transmission; however, there has never been direct detection of ILTV from air samples. We aimed to optimize a sampling system for the detection of airborne ILTV in poultry sheds.

**Results:**

Poultry farms with a known outbreaks of ILT were used for detection of airborne ILTV. Infected chickens were verified by detection of ILTV nucleic acid in feather shafts with all farms being positive. Using a liquid cyclonic impinging device, it was found that recovery and detection of airborne ILTV was possible in alkaline PEG buffer. Additional sampling was performed at different heights to determine the presence of ILTV in the air. In farm 3, all three air samples at both heights were positive for ILTV while at farm 2 only one sample at 45 cm was positive. We envisaged in the future air sampling will be able to detect and track potential transmission of ILTV both inside and outside of the poultry shed.

## Introduction

The causative agent of infectious laryngotracheitis (ILT) is gallid herpesvirus 1, a highly contiguous virus that is characterized by severe dyspnea, coughing, and rales [1]. ILT infection has a significant economic impact on the poultry industry across the world due to reduce egg production and large mortality rate (up to 70%) [2]. Live attenuated vaccines, and biosecurity measures are currently the most effective control measures for ILT [2]. However, outbreaks of ILT occur regularly in Australia and recently, this resulted in novel strains due to recombination of the vaccine strains [3, 4].

Due to the substantial increase in the number of outbreaks of ILT in broiler farms in the Mornington Peninsula area of Victoria raised the concerns about the route of transmission of gallid herpesvirus 1. Potential sources of gallid herpesvirus 1 infection between poultry farms are infected chickens, contaminated litter, dust, drinking water, fomites and darkling beetles, which infect poultry through ocular and respiratory routes [2]. However, there were concerns that transmission between farms were occurring by being air. A single report suggests that airborne transmission is the likelihood of infection is ten times higher when within the vicinity of a clinically infected farm and within the vicinity of market gardens that apply raw poultry manure as fertiliser however, no direct testing of air was performed [5]. It has also been shown that relatively high levels of infectious gallid herpesvirus 1 are carried in dust which could result in increased airborne transmission [6]. Some other viral poultry diseases are transmitted by air, and thus it is a priority to determine if the wind also spreads gallid herpesvirus 1. Here, we report a sampling system for the detection of airborne gallid herpesvirus 1 in commercial poultry sheds.

## Main text

### Materials and methods

#### Farm identification, selection and definitions

In this study, three case farms were selected throughout March and April of 2017 from a list of broiler farms located in the Mornington Peninsula region, Victoria. The flocks were comprised of mixed-sex of Cobb and Ross broiler breeds between 36–50 days of age. Upon the onset of an outbreak of ILT in a poultry farm based on clinical diagnosis, the company veterinarian or service person notified the investigators. As soon as ILT case farms had been identified, an investigator visited the farm and collected air samples within 24 h.

#### Air sampling procedure

**Fig. 1:**
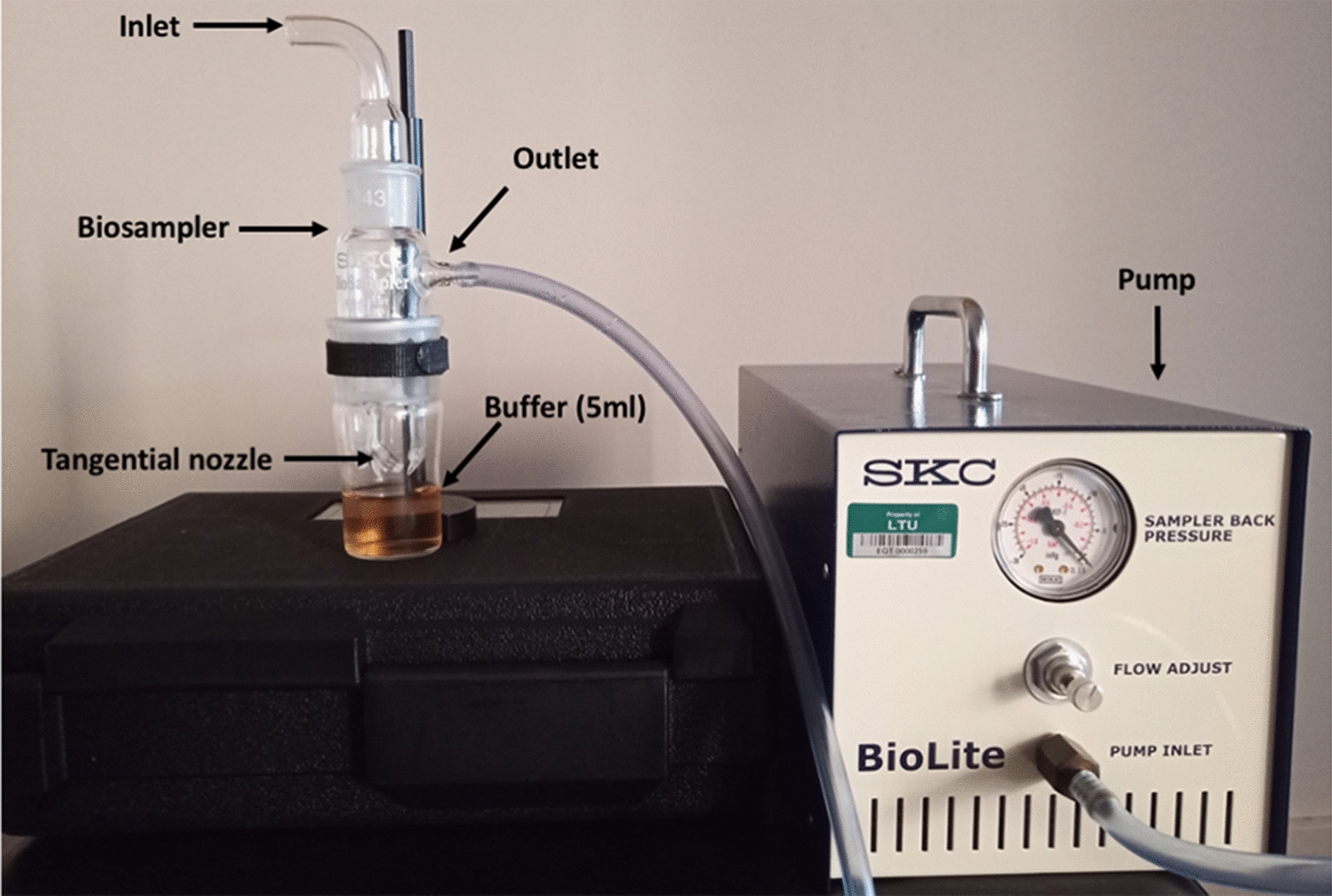
SKC Biosampler experimental setup. The glass Biosampler was assembled with glass collection vessel containing 5 ml of buffer in which the pump was attached to drive air through the collection buffer using the specialized tangential nozzle. The Biosampler were placed at different heights for the collection of airborne material

All air samples were collected using the SKC Biosampler^®^; a liquid cyclonic impinging device (SKC Inc, USA) and BioLite pump (SKC Inc, USA) as shown in Fig. 1. Briefly, the collection vessel contained 5 ml of alkaline polyethene glycol (PEG) solution (60% v/v PEG 200 (Sigma-Aldrich, USA), 20 mM KOH, pH 13.5) or phosphate buffer saline (PBS) (8.1 mM Na_2_HPO_4_, 137 mM NaCl, 1.4 mM KH_2_HPO_4_ and 2.6 mM KCl) in which material from the air was trapped. The air sampler was used for 20 min in three different locations within the shed at the height of either 45 cm or 120 cm off the floor at a rate of 12.5 L per min placed on card table. In between sampling, the glass collection vessel was disinfected with 80% ethanol. Each sample was transferred from the collection vessel to a clean sterile 15 ml conical centrifuge tubes for transport and storage in the laboratory at –20 °C.

#### DNA extraction of feather samples

Feather samples were gathered randomly off the floor inside the selected sampling shed. Feathers were collected and stored in 50 ml conical centrifuge tubes and stored at –20 °C until processing. Approximately 5 mm of each collected feather shaft was cut off and were divided into groups of three, with four shafts per group. Total DNA was extracted from feathers tips using the Mouse Direct PCR Kit (Biotool, USA) following the manufacturer’s instructions.

#### DNA purification of air and feather samples

DNA purification from air and feather samples were performed as described by Li and Sheen with the following modifications [7]. Briefly, 500 μl of DNA binding solution (6 M NaI) is added to 300 μl of extracted DNA feather sample to which 10 μl of 100 mg/ml silica dioxide (Sigma, USA) was added, vortexed then incubated at room temperature for 2 min. Samples were centrifuged for 10 s, and the supernatant removed. Silica matrix was then washed with 500 μl of washing solution (50% v/v ethanol, 10 mM Tris–HCl pH 7.5, 100 mM NaCl and 1 mM ethylenediaminetetraacetic acid (EDTA), centrifuged at 13,000x*g*, discarded supernatant and repeated once. Silica matrix was resuspended in 30 μl of sterile water and incubated at 70 °C for 2 min. The samples were centrifuged at 16,000x*g* for 2 min and eluted DNA transferred to a fresh tube and stored at −20 °C. A similar purification method was used for the air sample except the starting volume was 2.5 ml to which 3 ml of DNA binding solution and 100 μl of 100 mg/ml silica dioxide (Sigma, USA) was added. DNA was eluted by the addition of 100 μl of sterile water.

#### PCR for detection of Gallid herpesvirus 1

Conventional PCR was used for the detection of gallid herpesvirus 1 by the presence of the thymidine kinase (TK) gene, as described by Mahmoudian et al. (2011), with the following modifications [8]. Reactions were carried out in a final volume of 25 µL and amplified in a C-Master GT thermal cycler (Dynamica, Australia). The following amplification conditions consisted of an initial denaturation step of 94 °C for 3 min, 94 °C for 15 s, 60 °C for 45 s, 72 °C for 150 s, repeated for 35 times with a final extension at 72 °C for 3 min. Amplicon size was checked by agarose electrophoresis migration by loading directly on 1% (w/v) agarose (Lonza, USA) gel, prepared as per manufacturer’s instructions, with the addition of 0.5 µL of Sybr^®^ Safe DNA Gel Stain (Life Technologies, USA). The gel was run at 110 V for 45 min and imaged using the GelDoc^TM^ XR + (BioRad, USA) instrument and software.

### Results

**Fig. 2:**
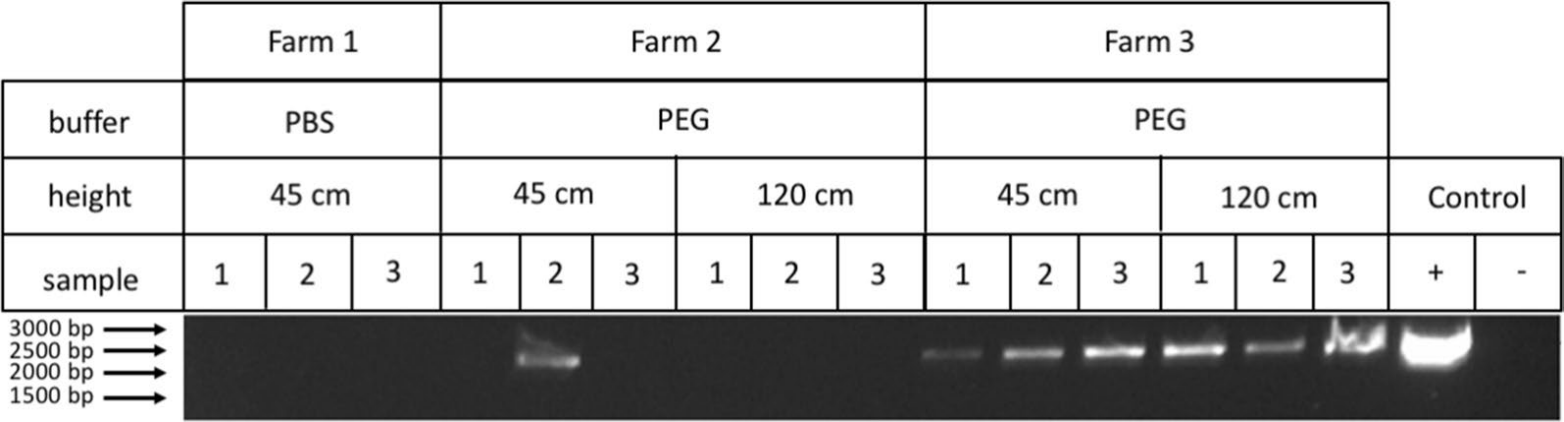
Identification of gallid herpesvirus 1 from air samples by PCR. Air samples collected from various farms at different heights and buffers were analyzed for the presence of gallid herpesvirus 1 by PCR. The amplified product was analyzed on a 1% (w/v) agarose gel. A single band is shown that corresponds to predicted amplicon containing the TK gene (2250 bp). The purified gallid herpesvirus 1 DNA from infected tissue was used as a positive control (+) template, and no amplification was seen in the negative (−), no template, control

**Fig. 3:**
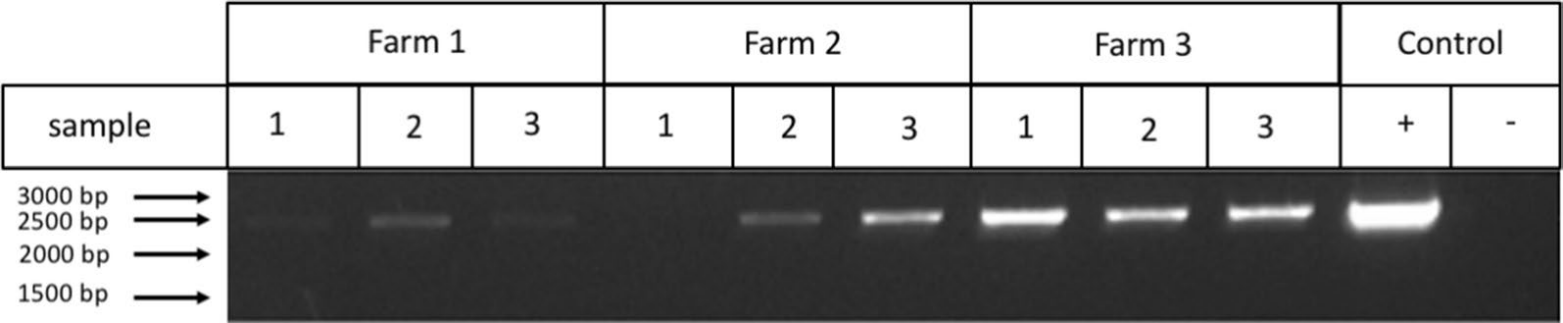
Identification of gallid herpesvirus 1 from feather shafts by PCR. Pools of feathers (n = 4) per sample were collected randomly from the floor of the poultry shed and presence of gallid herpesvirus 1 was determined by PCR. A single band is shown that corresponds to predicted amplicon containing the TK gene (2250 bp). The purified gallid herpesvirus 1 DNA from infected tissue was used as a positive control (+) template, and no amplification was seen in the negative (−), no template, control

Air samples in farm 1 were initially collected using a PBS buffer; however, the presence of gallid herpesvirus 1 was not detected suggesting the possibility this buffer is unsuitable for collection of viral samples (Fig. 2) (Additional file 1). However, the use of alkaline PEG as a collection buffer in farms 2 and 3 allowed the detection of gallid herpesvirus 1 by amplification of the TK gene (2250 bp amplicon) on new farms (Fig. 2). Sampling occurred at two heights with all sample sites and heights in farm 3 being positive for gallid herpesvirus 1 while only one site in farm 2 at 45 cm off the floor was positive (Fig. 2). All farms that were sampled had reported outbreaks of ILT and thus to confirm the presence of ILTV infected chickens on farms; a non-invasive method was used [9]. Feathers were collected off the ground and DNA extracted from shafts. The presence of gallid herpesvirus 1 was seen at all farms with Farm 3 showing the highest level of infection (Fig. 3) (Additional file 1).

### Discussion

We have developed a sampling system for the detection of airborne gallid herpesvirus 1 by PCR. The initial use of PBS buffer to collect airborne material was unsuccessful which was a surprise as has been used in the past as a collection media in the successful isolation of airborne Porcine Reproductive and Respiratory Syndrome Virus (PRRSV) and influenza H1N1 [10, 11]. However, this was done in conjunction with other solutions such as ethylene glycol, bovine serum albumin (BSA) and activated charcoal [11]. The use of alkaline PEG solution as sample buffer was successful in detecting the presence of gallid herpesvirus 1 which may be due to the fact this solution causes lysis of majority of microbes and inhibition of enzymes due to high pH which would preserve nucleic acid [12]. However, the use of this buffer excludes testing whether the detected nucleic acid was infectious. On farm 3 both measured heights 45 cm and 120 cm recorded positive samples in comparison with farm 2, which only had one positive sample at 45 cm high. This is approximately the same level as a chicken’s mouth. It has been shown that relative humidity (RH) can affect the airborne transmission of influenza virus, as demonstrated in the guinea pig model with deficient transmission at mid-range and very high RH [13]. Ideally the RH should be between 50 and 70% in poultry shed (https://www.poultryventilation.com/node/4925) however the RH in each shed at farms 2 and 3 was not directly measured it was observed that on day of collection farm 2 had high humidity (> 70%). In contrast, on farm 3 it had low humidity (< 50%) suggesting that the humidity could be another factor that affects the transmission of gallid herpesvirus 1 by air.

To confirm the presence of gallid herpesvirus 1 on farms, feather shafts were used as a source of our DNA. This method is particularly beneficial as they are easy to collect, non-lethal for the bird, therefore useful for monitoring purposes. The majority of feather samples were positive for the presence of gallid herpesvirus 1, except for one sample in farm 2. On farm 2 it appears to have a low level of infection which may be why only one of the air samples from this farm returned a positive result.

In the future, this method will allow us to investigate whether transmission of ILT between poultry farms could occur via the air. In addition, these results will enable greater insight into the use of air sampling for the detection of other poultry viral diseases. This information will help in the deployment of air sampling and feather collection as means for a non-invasive biosecurity surveillance for the poultry industry.

## Limitations

There are several limitations of this current study. (1) The current use of alkaline PEG does not allow for isolation and viability testing of the collected virus; thus use of PBS is required. Further work is required to develop a collection buffer to allow isolation and viability testing of gallid herpesvirus 1. (2) It appears humidity can play a role in the ability of gallid herpesvirus 1 to be airborne and direct measurements of humidity in sheds are required. (3) There were no molecular tests performed to distinguish the collected gallid herpesvirus 1 virus from vaccine strains and field isolates in this study. (4) The need to use quantitative PCR (qPCR) to measure v.

## Supplementary information

**Additional file 1:** The raw electrophoresis results for the identification of gallid herpesvirus 1 by PCR from collected air samples.

## Data Availability

The information supporting the conclusions of this article is included in the article.

## Abbreviations

ILT: Infectious laryngotracheitis
ILTV: Gallid herpesvirus 1
PCR: Polymerase chain reaction
PEG: Polyethene glycol
PBS: Phosphate buffer saline
DNA: Deoxyribonucleic acid
EDTA: Ethylenediaminetetraacetic acid
TK: Thymidine kinase
PRRSV: Porcine Reproductive and Respiratory Syndrome Virus
RH: Relative humidity
