# Supplementary material for: Air sampling for detection of infectious laryngotracheitis (ILT) in commercial poultry flocks
Source: BMC Res Notes. 2020 Dec 9;13:556. doi: 10.1186/s13104-020-05399-2 (PMC7727125; doi:10.1186/s13104-020-05399-2)
Supplement: Supplementary file 1 — Additional file 1: The raw electrophoresis results for the identification of gallid herpesvirus 1 by PCR from collected air samples. [file 13104_2020_5399_MOESM1_ESM.pdf]

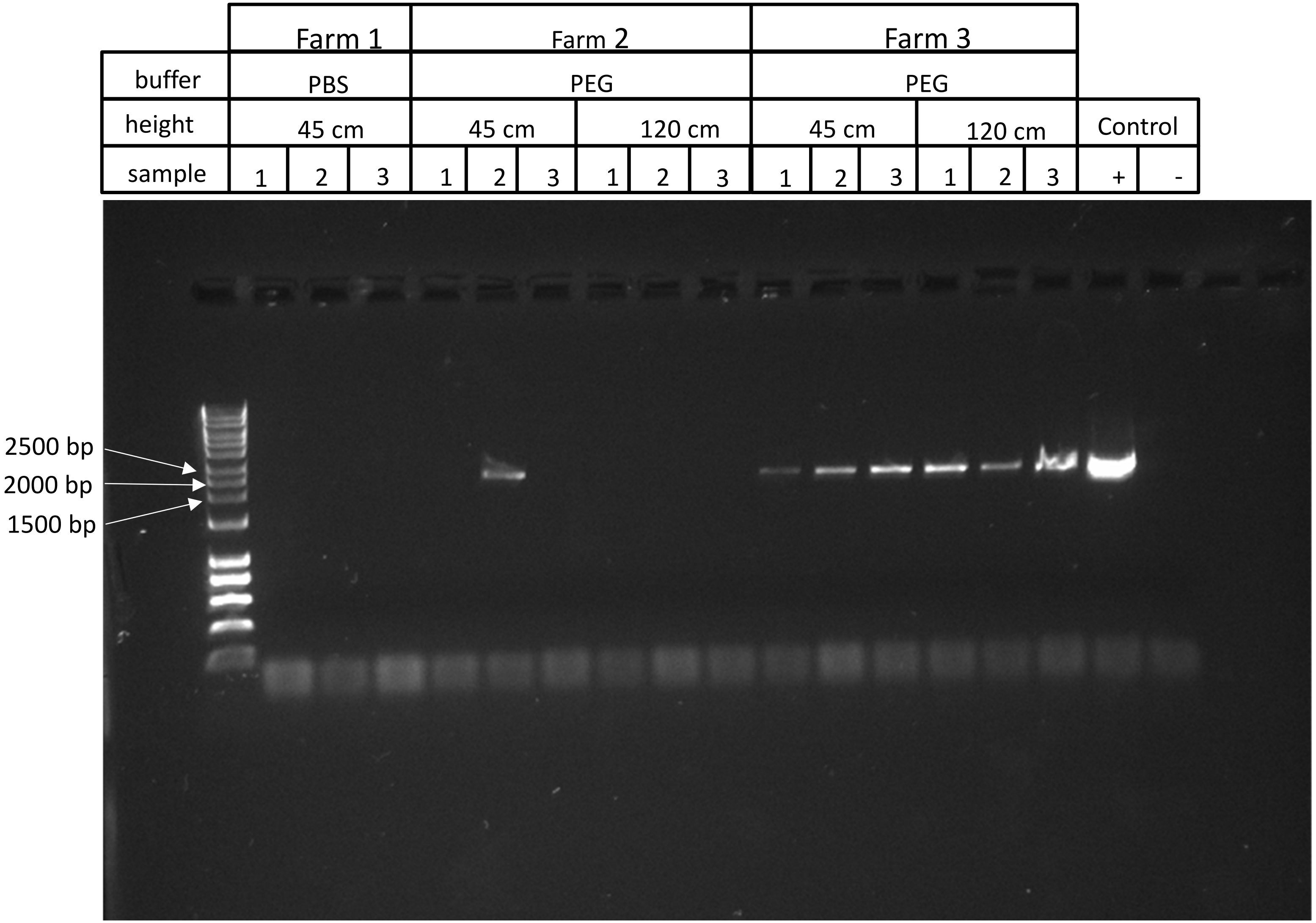

Figure 2. **Identification of gallid herpesvirus 1 from air samples by PCR.** Air samples collected from various farms at different heights and buffers were analyzed for the presence of gallid herpesvirus 1 by PCR. The amplified product was analyzed on a 1% (w/v) agarose gel. A single band is shown that corresponds to predicted amplicon containing the TK gene (2250bp). The purified gallid herpesvirus 1 DNA from infected tissue was used as a positive control (+) template, and no amplification was seen in the negative (-), no template, control

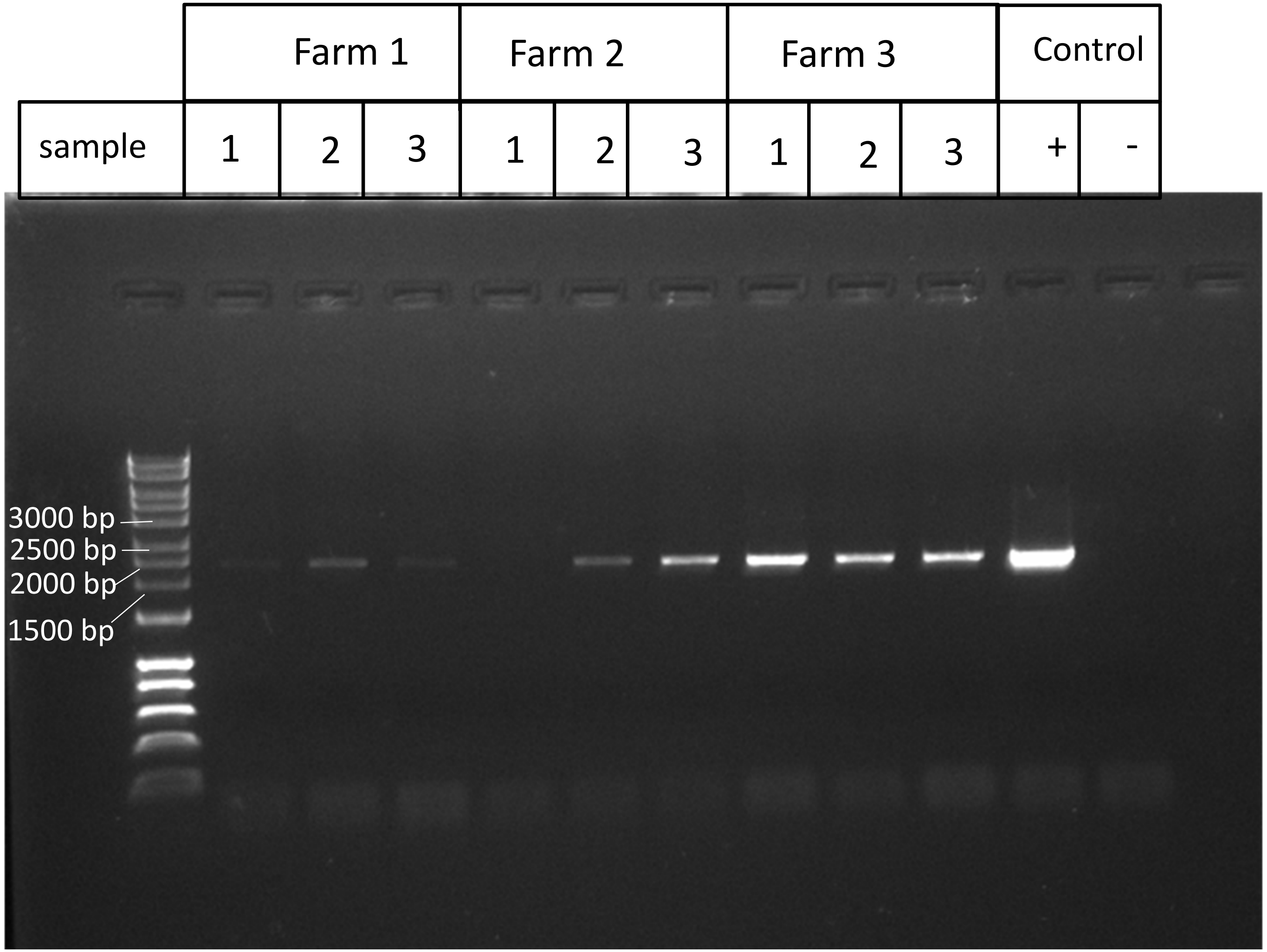

Figure 3. **Identification of gallid herpesvirus 1 from feather shafts by PCR.** Pools of feathers (n=4) per sample were collected randomly from the floor of the poultry shed and presence of gallid herpesvirus 1 was determined by PCR. A single band is shown that corresponds to predicted amplicon containing the TK gene (2250bp). The purified gallid herpesvirus 1 DNA from infected tissue was used as a positive control (+) template, and no amplification was seen in the negative (-), no template, control.
